# Knowledge, attitudes and behaviours toward COVID-19: A cross-sectional survey among Nigerian University students

**DOI:** 10.4102/hsag.v27i0.1725

**Published:** 2022-05-27

**Authors:** Philemon D. Shallie, Firoza Haffejee

**Affiliations:** 1 Department of Basic Medical Sciences, Faculty of Health Sciences, Durban University of Technology, Durban, South Africa; 2 Department of Anatomy, Faculty of Basic Medical Sciences, Olabisi Onabanjo University, Sagamu, Nigeria

**Keywords:** attitudes, behaviours, COVID-19, knowledge, Nigerians

## Abstract

**Background:**

The effect of the highly contagious coronavirus disease 2019 (COVID-19) began in Wuhan, Hubei Province, China, from which it spread worldwide. In Nigeria, to curb the spread of the virus, the government elected to close public places, halt the general use of public transportation, enforce isolation and manage infected persons.

**Aim:**

This study evaluated Nigerian university students’ knowledge, attitudes and behaviour (KAB) towards COVID-19.

**Setting:**

This was an online survey of Nigerian university students.

**Method:**

A cross-sectional study was conducted among 1268 respondents aged 16 to 60 who completed the survey questionnaire. The respondents’ demographic data and KAB toward COVID-19 were collected, allocated and scored based on specific stratified divisions. Data were analysed using student’s *t*-test, analysis of variance and logistic regression analysis.

**Results:**

The respondents demonstrated good knowledge of COVID-19, with a mean knowledge score of 78.7%; this positively influenced their attitude and behaviour scores (84.1% and 72.3%, respectively). Multiple linear regression analysis showed that 98.9% of the variance associated with poor knowledge is explained by gender (98.9%), age (97.3%), education (97.3%), occupation (97.2%) and marital status (91.4%).

**Conclusion:**

The respondents had a positive attitude and satisfactory compliance with safety practices required to curb the spread of the virus. Nevertheless, there is a need to intensify health education campaigns targeting all Nigerians, especially the less educated, via community outreach programmes using local languages.

**Contribution:**

The findings of this study demonstrate the imperative role of the knowledge of COVID-19 in curbing the spread of the infection via improved attitudes and positive behaviours in compliance with safety practices.

## Introduction

The cases of unexplained pneumonia reported in Wuhan, China, in December 2019 prompted swift action by researchers and the Chinese government to control the epidemic (Sun et al. 2020). A month later, precisely on 12 January 2020, the new virus was tentatively named coronavirus 2019 (2019 – nCoV) by the World Health Organization (WHO). The virus reached a pandemic proportion on 30 January 2020 and was declared a public health emergency of international concern by the WHO (2020). The disease triggered by 2019 – nCoV was formally named on 11 February 2020 as coronavirus disease 2019 (COVID-19) and 2019 – nCoV as coronavirus 2 of the severe acute respiratory syndrome (SARS-CoV-2) respectively by the WHO and the International Committee on Virus Taxonomy (Wang et al. 2020). The COVID-19 clinical symptoms include fever, fatigue, dry cough, malaise and breathing difficulty (Sun et al. 2020).

This highly contagious virus’ ravaging effect began in Wuhan, Hubei Province, China and then around the world (Reuben et al. 2020; Wang et al. 2020; Zhong et al. 2020) except Antarctica (Rahman & Bahar 2020). The rate of infection and spread among the regions and countries of the world was on the rise. Therefore, the COVID-19 global lockdown was introduced to curb the spread of the virus and ‘flatten the curve’ of the pandemic (Onyeaka et al. 2021). As a result, by April 2020, more than 3.9 billion people, about half of the world’s population, were subjected to lockdown, having been compelled to stay at home by their governments (Levenson 2020).

Like the rest of the world, Africa began enforcing partial or total closures of their borders, shutting down schools, prohibiting public congregations, enforcing other social distancing measures and announcing a state of emergency. The African lockdown was swiftly implemented in sub-Saharan Africa, probably due to its previous pandemic experience (Shallie & Haffejee 2021).

Hospitals in Nigeria’s commercial capital Lagos struggled to manage the influx of COVID-19 patients (Daily-Briefings 2020). In Nigeria, the public opinion was that COVID-19 is a ‘big man disease’, that is, disease of wealthy people (Akhaine 2020). This misconception, coupled with urban ghettos, high population, poor and scarce potable water and a weak healthcare system, made it difficult to introduce hygiene and other public health policies for restraining the coronavirus (Reuben et al. 2021).

The fight against COVID-19 is ongoing, with subsequent waves of high infection and new virus strains. To control the coronavirus, people need to strictly adhere to the standard infection control measures. Adherence and compliance are influenced by people’s knowledge, attitudes and behaviours (KAB) to prevent infection of the virus (Ajilore, Atakiti & Onyenankeya 2017; Tachfouti et al. 2012). The lockdown significantly impacted people’s daily social routine, characterised by longer solitary time at home, resulting in decreased interpersonal human contacts (Saladino et al. 2020). It is unknown how strict measures to control the spread of COVID-19 would affect the people, especially the youth, who constitute a large proportion of the population. A previous study on knowledge, attitudes and perceptions of Ebola Virus Disease (EVD) among college students in Nigeria assessed college students’ adherence to EVD PSAs. The findings generally suggest that students are aware of the causes, symptoms and treatments of EVD and are willing to take preventive actions to avoid contracting or spreading the disease (Ajilore et al. 2017). Furthermore, there is an association between access to EVD public service announcements (PSAs) and a high level of knowledge of EVD among college students (Ajilore et al. 2017). As the authors advance, this positive correlation between understanding the disease and accessibility to PSA has significant ramifications for prevention education initiatives for both EVD and other infections such as COVID-19. Proactive communication strategies through sustained PSAs and other sources of health-related information sharing are required to dispel the widespread idea that these illnesses will never recur in Nigeria. This advocacy will forestall the likelihood of abandoning the preventive actions and habits acquired or relapse into unhealthy practices. The authors, therefore, investigated the KAB towards COVID-19 among students of a Nigerian tertiary educational institution.

## Research methods

### Study design

This cross-sectional quantitative study was carried out from 20 August to 20 October 2020. A quantitative research approach focuses on counting data, and this method is used when a phenomenon is studied in terms of quantity. Kothari (2004) stated that this is the best design to evaluate specific answers to particular questions, which in this context consisted of the KABs towards COVID-19 (Kothari 2004). A self-administered online survey using Google Forms was used for collecting the data.

### Study setting

The survey was distributed through existing university-based student WhatsApp groups of students’ associations and various courses at different levels of study, which were randomly chosen by a ballot. Snowball sampling was subsequently used to recruit more participants. This facilitated easy recruitment of more participants who were referred by those who entered the study as described above.

Consent was obtained on the Google Form, from all those willing to participate, prior to them answering the questions. The student association leaders and course leaders served as key informants who posted the questionnaires on their WhatsApp groups. As the minimum age requirement for admission into higher educational institutions in Nigeria is 16 years, students 16 years and over were invited to participate in the study. In Nigeria, parental consent is not required for persons aged 13 and above as per guidelines by the Federal Ministry of Health (FMoH) and the National Health Research Ethics Committee of Nigeria (NHREC) (FMoH 2014; NHREC 2016).

### Study population

The study was conducted among 1268 registered male and female students, aged 16 years or older at a university in Ogun State, which has a total population of 27 000 students.

### Questionnaire development

After obtaining permission from the authors, Zhong et al.’s (2020) instrument was adapted for this study. This questionnaire comprised of two parts, namely, sociodemographic characteristics and KAB, with 12 questions on knowledge of COVID-19: four regarding clinical presentations, three on transmission and five regarding prevention and control of COVID-19. These questions were answered on a 3-point scale with true, false, and ‘I don’t know’ options. A correct answer was assigned one mark, and an incorrect or unknown answer was assigned zero. The cumulative total knowledge score points could thus potentially range from a minimum of 0 to 12 as the highest possible score, with a higher score indicating better knowledge of COVID-19. Attitudes toward COVID-19 were measured using three questions about control measures, such as social distancing, wearing a mask and regular washing or sanitising of hands to curb the spread of COVID-19.

The content validity of the questionnaire was ensured by conducting a pilot study among 30 students. The dichotomous scale (knowledge subscale) and Cronbach’s α coefficient for the four-point Likert scale (attitude and practices subscales) were evaluated for internal consistency and reliability using the Kuder-Richardson-20 (KR-20). Satisfaction was determined at KR-20 coefficient of ≥ 0.50 and Cronbach’s α coefficient of ≥ 0.70.

Individual scores for KAB of different participants were calculated. These were correlated with the various demographic characteristics using independent sample *t*-tests, one-way analysis of variance (ANOVA) or chi-square tests, as appropriate. To identify factors associated with knowledge, we performed multivariate regression analyses on all demographic variables as independent variables and the knowledge score as the outcome variable. SPSS (version 20) was used to analyse the data and determine statistical significance at *p* < 0.05.

### Ethical considerations

Ethical approval for this study was obtained from the Institutional Research Ethics Committee at Olabisi Onabanjo University, Sagamu, Nigeria (No. OOU/IREC/20/57). Participation in the study was voluntary and all participants were required to read the letter of information and provide consent. No person was coerced into participating in the study.

## Results

### Sociodemographic characteristics

A total of 1268 respondents completed the survey questionnaire. Sixty-two questionnaires were excluded as they were incomplete. The age of the respondents ranged between 16–60 years, with 97.45% (*n* = 1236) under the age of 30 years, 56.8% (*n* = 720) were women, 98.8% (*n* = 1252) were either undergraduate students or held a bachelor’s degree or above. The majority 74.1% (*n* = 940) were Christian, never married 94% (*n* = 1192) and lived in the southwestern region of the country 80.4% (*n* = 1020). Other sociodemographic characteristics are shown in Table 1.

**TABLE 1:** Demographic characteristics of participants and knowledge score of COVID-19 by demographic variables.

Characteristics	Number of participants (%)		Knowledge score		*t*/F	*P*
	*N*	%	Mean	± Standard deviation		
**Gender**	-	-	-	-	0.1759	> 0.05
Female	720	56.8	9.8	±2.4	-	-
Male	548	43.2	9.6	±2.3	-	-
**Age Group**	-	-	-	-	9.631	< 0.0001
16 to 20	488	38.5	9.8	±2.4	-	-
21 to 25	588	46.4	9.7	±2.2	-	-
26 to 30	124	9.8	9.5	±1.9	-	-
31 to 40	36	2.8	9.9	±2.9	-	-
41 to 50	8	0.6	6.5	±4.8	-	-
51 and above	24	1.9	7.9	±4.3	-	-
**Marital Status**	-	-	-	-	1.551	< 0.05
Never-Married	1192	94.0	9.2	± 0.9	-	-
Married	76	6.0	10.2	± 0.7	-	-
**Religion**	-	-	-	-	0.1339	> 0.05
Christianity	940	74.1	9.7	±2.3	-	-
Islam	324	25.6	9.7	±2.1	-	-
Traditionalist	4	0.3	9.0	±5.4	-	-
**Education**	-	-	-	-	1.025	> 0.05
Graduate	256	20.2	9.8	±2.3	-	-
Postgraduate	76	6.0	9.9	±2.4	-	-
Undergraduate	936	73.9	9.6	±2.3	-	-
**Occupation**	-	-	-	-	0.0492	> 0.05
Government employee	72	5.7	10.1	±3.1	-	-
Private sector worker	60	4.7	10.1	±2.1	-	-
Self employed	120	9.5	9.9	±2.7	-	-
Student	924	72.9	9.7	±2.3	-	-
Unemployed	92	7.3	10.0	±2.4	-	-
**Residential Region**	-	-	-	-	0.3482	> 0.05
Northcentral	64	5.0	9.3	±3.1	-	-
Northeast	40	3.2	8.7	±3.2	-	-
Northwest	44	3.5	9.6	±2.3	-	-
Southeast	28	2.2	10.0	±3.1	-	-
Southsouth	72	5.7	9.2	±2.2	-	-
Southwest	1020	80.4	9.8	±2.2	-	-

### The influence of sociodemographic characteristics on coronavirus disease 2019 knowledge

The correct answers mean score rates of the 12 questions on the COVID-19 knowledge questionnaire ranged between 6.5 and 10.2 (Table 1). The mean COVID-19 knowledge score was 9.44 ± 0.79, indicating an overall 78.7% (9.44/12*100) correct score rate on this knowledge test. The knowledge scores differed significantly across all age groups (*p* < 0.0001) and marital status (*p* < 0.05) (Table 1). There were no significant differences across other demographic characteristics.

Multiple linear regression analysis showed that 98.9% of the variance associated with poor knowledge is gender. The female gender (β: 4.99, *p* < 0.009) had a significantly higher association with poor (low) knowledge score compared to the male gender (β: 2.97, *p* > 0.05); the second factors associated with poor knowledge score were both age and education with 97.3%. The age group of 16–30 years (β: −7.05, *p* < 0.0001) was significantly associated with poor knowledge score compared to age group 31 and above (β: −0.462, *p* > 0.05), whilst bachelor’s degree level of education (β: −12.72, *p* < 0.01) had a significantly lower association with a poor knowledge score compared to postgraduate degree level of education (β: 5.09, *p* > 0.05); occupation with 92.2% of the variance was the third factor associated with a poor knowledge score. Those who were self-employed had a significantly lower association with poor knowledge scores (β: 0.298, *p* > 0.05). Finally, marital status showed the least variance associated with poor knowledge scores. Married respondents (β: 5.68, *p* < 0.0001) had a significantly higher association with a decrease in knowledge scores compared to never-married respondents (β: 0.218, *p* > 0.05); 97.3% of the variance associated with poor knowledge score is explained by education (Table 2).

**TABLE 2:** Results of multiple linear regressions on factors associated with poor COVID-19 knowledge.

Variable	F	Sig.	Adjusted R Square	Coefficient	Standard error	*t*	*P*
**Gender**	479.40	0.000	0.989	-	-	-	-
Female	-	-	-	4.99	1.42	3.31	0.009
Male	-	-	-	2.97	1.46	2.03	0.073
**Age-group**	198.03	0.000	0.973	-	-	-	-
16-30 years	-	-	-	−7.052	0.692	−10.198	0.000
31 years and above	-	-	-	−0.462	0.364	−1.270	0.236
**Marital status**	59.08	0.000	0.914	-	-	-	-
Never-married	-	-	-	0.218	0.027	0.234	0.820
Married	-	-	-	5.681	0.946	8.068	0.000
**Education**	134.84	0.000	0.973	-	-	-	-
Bachelor degree level	-	-	-	−12.717	3.33	−3.788	0.005
Postgraduate degree level	-	-	-	5.093	3.57	1.525	0.065
**Occupation**	96.18	0.000	0.972	-	-	-	-
Unemployed	-	-	-	0.298	1.24	0.241	0.817
Payed-Employment	-	-	-	0.266	2.50	0.106	0.919
Self-Employment	-	-	-	−1.622	1.79	−0.909	0.002
Students	-	-	-	−6.787	1.38	−4.92	0.394

### The influence of sociodemographic characteristics on attitudes toward coronavirus disease 2019 control

There was a very high positive attitude (agree) amongst the respondents toward the control measures to curb the spread of COVID-19. Most respondents, 88.2% (*n* = 967), agreed that COVID-19 would be successfully controlled. Rates of reporting ‘disagree’ (negative attitudes) and ‘I don’t know’ (undefined attitude) were 3.4% (*n* = 37) and 8.4% (*n* = 92) respectively. Of the respondents, 80.0% (*n* = 877) were confident that COVID-19 would be curbed, whilst 6.8% (*n* = 75) of respondents disagreed and 13.2% (*n* = 145) responded that they did not know. The positive attitude toward curbing the spread of COVID-19 was significantly high in all the parameters assessed except for age group over 40 years, traditional religious believers and respondents who resided in the South Eastern region of Nigeria. In addition, only 55.7% (*n* = 611) of the respondents had a positive attitude toward using personal protective equipments (PPEs) against COVID-19. In comparison, 44.3% (*n* = 486) did so out of fear of a penalty, and 7.4% (*n* = 81) had no confidence that PPEs would protect against COVID-19 infection. The attitude toward using PPEs varied significantly across the female gender, age group 31 to 40, those who were never married, postgraduates, undergraduates, Christians, paid employees, unemployed students, residents of North Central, South South, and South East regions of the country. The respondents with a negative attitude towards controlling COVID-19 had significantly poorer knowledge of COVID-19 (*p* < 0.05) than those with a positive attitude (Table 3). Multiple logistic regression analysis showed that only COVID-19 knowledge score 1.111 (0.988, 1.248), *p* < 0.05, was significantly associated with negative attitudes (Table 4).

**TABLE 3:** Attitudes towards COVID-19 by demographic variables.

Characteristics	Attitudes, *n* (%) or mean (standard deviation)															
	A1: final success in controlling						A2: confidence of winning						A3: Penalty induced compliance			
	Yes		No		Don’t know		Yes		No		Don’t know		Yes		No	
	*N*	%	*N*	%	*N*	%	*N*	%	*N*	%	*N*	%	*N*	%	*N*	%
**Gender**																
Female	612	86.9	16	2.3	76	10.8****	580	82.4	48	6.8	76	10.8****	216	42.2	296	57.8*
Male	484	88.3	20	3.6	44	8.0****	428	78.1	44	8.0	76	13.9****	320	46.5	368	53.5
**Age Group**																
16 to 20	420	87.5	8	1.7	52	10.8****	368	76.7	36	7.5	76	15.8****	204	44.3	256	55.7
21 to 25	524	90.3	16	2.8	40	6.9****	484	83.4	40	6.9	56	9.7****	268	47.9	292	52.1
26 to 30	92	74.2	12	9.7	20	16.1****	96	77.4	12	9.7	16	12.9****	48	42.9	64	57.1
31 to 40	36	100.0	0	0.0	0	0.0***	36	100.0	0	0.0	0	0.0***	4	11.1	32	88.9**
41 to 50	4	100.0	0	0.0	0	0.0	4	50.0	0	0.0	4	50.0	4	50.0	4	50.0
51 and above	20	100.0	0	0.0	0	0.0**	20	83.3	4	16.7	0	0.0**	8	33.3	16	66.7
**Marital status**																
Married	80	95.2	0	0.0	4	4.8***	72	85.7	8	9.5	4	4.8****	32	42.1	44	57.9
Never married	1016	86.4	36	3.1	124	10.5****	936	79.9	84	7.2	152	13.0****	504	45.0	616	55.0*
**Education**																
Graduate	224	87.5	4	1.6	28	10.9****	212	82.8	16	6.3	28	10.9****	120	50.0	120	50.0
Postgraduate	68	89.5	4	5.3	4	5.3****	68	89.5	4	5.3	4	5.3****	20	27.8	52	72.2*
Undergraduate	792	87.2	28	3.1	88	9.7****	716	78.9	72	7.9	120	13.2****	392	44.7	484	55.3*
**Religion**																
Christianity	812	87.5	28	3.0	88	9.5****	724	78.0	80	8.6	124	13.4****	380	43.0	504	57.0**
Islam	280	87.5	8	2.5	32	10.0****	280	86.4	12	3.7	32	9.9****	152	48.7	160	51.3
Traditionalist	4	100.0	0	0.0	0	0.0	8	100.0	0	0.0	0	0.0	4	100.0	0	0.0
**Occupation**																
Government employee	68	94.4	4	5.6	0	0.0****	64	88.9	8	11.1	0	0.0****	20	27.8	52	72.2*
Private sector worker	52	86.7	4	6.7	4	6.7***	48	80.0	4	6.7	8	13.3***	20	35.7	36	64.3*
Self employed	100	86.2	4	3.4	12	10.3***	84	72.4	8	6.9	24	20.7***	68	63.0	40	37.0
Student	812	88.3	20	2.2	88	9.6****	740	80.4	68	7.4	112	12.2****	392	44.3	492	55.7*
Unemployed	64	72.7	4	4.5	20	22.7***	72	81.8	4	4.5	12	13.6***	40	47.6	44	52.4
**Region of Resident**																
Northcentral	52	81.3	0	0.0	12	18.8***	40	62.5	8	12.5	16	25.0***	20	33.3	40	66.7*
Northeast	36	90.0	4	10.0	0	0.0**	32	80.0	4	10.0	4	10.0**	16	40.0	24	60.0
Northwest	44	100.0	0	0.0	0	0.0***	44	100.0	0	0.0	0	0.0***	24	54.5	20	45.5
Southeast	20	71.4	4	14.3	4	14.3*	12	42.9	4	14.3	12	42.9	16	57.1	12	42.9
Southsouth	64	88.9	4	5.6	4	5.6***	60	83.3	4	5.6	8	11.1**	56	77.8	16	22.2*
Southwest	852	87.3	28	2.9	96	9.8****	796	81.6	64	6.6	116	11.9****	392	42.2	536	57.8**
COVID-19 knowledge score	9.6	2.1	9.1	2.1	8.1	2.7**	9.8	2.2	9.1	2.4*	8.3	2.1***	9.4	2.2	9.7	2.1*

* , *p* < 0.05;

** , *p* < 0.01;

*** , *p* ≤ 0.001;

**** , *p* < 0.0001

**TABLE 4:** Results of multiple binary logistic regression analysis on factors associated with attitudes towards COVID-19.

Variable	OR	95%	CI	*P*
**Positive attitudes vs. Negative attitudes**				
Gender (Female vs. Male)	1.117	0.642	1.940	0.40
Age-group (16–30 vs. 31 years and above)	1.610	0.540	4.810	0.28
Religion (Christianity vs. Islam)	1.120	0.574	2.186	0.43
Marital status (Never-married vs. Married)	0.956	0.335	2.750	0.56
Graduate level education vs. Postgraduate level education	1.182	0.130	10.78	0.68
Occupation (Employed vs. Unemployed)	0.862	0.243	3.050	0.55
COVID-19 Knowledge score	1.111	0.988	1.248	0.04
**Positive attitudes vs. undefined attitudes**				
Gender (Female vs. Male)	1.251	0.580	2.720	0.36
Age-group (16–30 vs. 31 years and above)	1.403	0.296	6.660	0.46
Religion (Christianity vs. Islam)	0.762	0.275	2.110	0.40
Marital status (Never-married vs. Married)	1.398	0.174	11.150	0.61
Graduate level of education vs. Postgraduate level of education	0.552	0.060	5.114	0.48
Occupation (Employed vs. Unemployed)	1.867	0.462	7.540	0.29
COVID-19 Knowledge score	0.981	0.326	2.559	0.59

OR, odds ratio; CI, confidence interval.

### The influence of sociodemographic characteristics on behaviours toward coronavirus disease 2019

Regarding behaviour, about 47.9% (*n* = 525) of the respondents had not visited any crowded place, although most respondents, 92.5% (*n* = 1014), wore masks when going out during the lockdown. The behaviour of the respondents differed significantly across sociodemographic groups (*p* < 0.05), except for the rates of going to a crowded place (Table 5). Multiple logistic regression analysis showed that only COVID-19 knowledge score 1.128 (1.004, 1.208; *p* < 0.05) was significantly associated with behaviour to prevent a possible infection (Table 6).

**TABLE 5:** Behaviours towards COVID-19 by demographic variables.

Characteristics	Practices, *n* (%) or mean (standard deviation)											
	P1: In recent days, have you gone to any crowded place?				P2: In recent days, have you worn a mask when leaving home				P3: Do you always wash your hands when you return home			
	Yesv		No		Yes		No		Yes		No	
	*N*	%	*N*	%	*N*	%	*N*	%	*N*	%	*N*	%
**Gender**												
Female	368	52.9	328	47.1	692	92.5	52	7.5***	356	50.6	348	49.4
Male	264	51.2	252	48.8	528	92.4	40	7.6***	252	46.0	296	54.0
**Age Group**												
16 to 20	208	44.8	256	55.2	464	93.1	32	6.9****	224	46.7	256	53.3
21 to 25	324	57.4	240	42.6*	576	91.7	48	8.3****	276	47.6	304	52.4
26 to 30	48	41.4	68	58.6	112	96.4	4	3.6**	68	54.8	56	45.2
31 to 40	24	66.7	12	33.3	36	88.9	4	11.1**	20	62.5	12	37.5
41 to 50	8	100.0	0	0.0*	12	66.7	4	33.3	4	50.0	4	50.0
51 and above	20	83.3	4	16.7*	24	83.3	4	16.7*	16	66.7	8	33.3
**Marital status**												
Married	56	73.7*	22	6.3	76	94.7	4	5.3*	48	63.2	28	36.8*
Never married	576	50.5	564	49.5	1148	92.3	88	7.7****	560	47.8	612	52.2
**Education**												
Graduate	144	61.0	92	39.0*	244	95.1	12	4.9***	132	49.3	136	50.7
Postgraduate	52	68.4	24	31.6*	76	78.9	16	21.1*	44	57.9	32	42.1
Undergraduate	424	47.7	464	52.3	888	92.8	64	7.2****	420	46.3	488	53.7
**Religion**												
Christianity	488	54.7	404	45.3	904	91.6	76	8.4****	440	47.4	488	52.6
Islam	140	44.3	176	55.7	312	94.9	16	5.1***	164	51.3	156	48.8
Traditionalist	4	100.0	0	0.0	4	100.0	0	0.0	4	100.0	0	0.0
**Occupation**												
Government employee	56	77.8	16	22.2*	72	77.8	16	22.2*	40	55.6	32	44.4
Private sector worker	40	66.7	20	33.3	60	93.3	4	6.7**	40	66.7	20	33.3
Self employed	56	51.9	52	48.1	108	96.3	4	3.7**	60	50.0	60	50.0
Student	436	48.9	456	51.1	900	92.9	64	7.1****	428	46.5	492	53.5
Unemployed	44	52.4	40	47.6	84	95.2	4	4.8**	40	45.5	48	54.5
**Region of Resident**												
North-Central	24	40.0	36	60.0	60	86.7	8	13.3**	40	62.5	24	37.5
North-East	28	70.0	12	30.0	40	80.0	8	20.0**	24	60.0	16	40.0
North-West	16	36.4	28	63.6	44	90.9	4	9.1**	28	63.6	16	36.4
South-East	12	42.9	16	57.1	28	85.7	4	14.3*	16	57.1	12	42.9
South-South	44	61.1	28	38.9	72	83.3	12	16.7**	36	50.0	36	50.0
South-West	492	52.3	448	47.7	952	95.0	48	5.0****	452	46.3	524	53.7*
COVID-19 knowledge score	9.5	2.2	9.6	2.3	9.7	2.1	8.3	2.6**	9.2	2.9	9.0	2.4

* , *p* < 0.05;

** , *p* < 0.01;

*** , *p* ≤ 0.001;

**** , *p* < 0.0001

**TABLE 6:** Results of multiple binary logistic regression analysis on factors associated with behaviours towards COVID-19.

Variable	OR	95%	CI	*P*
Gender (Female vs. Male)	0.765	0.472	1.242	0.17
Age-group (16–30 vs. 31 years and above)	0.537	0.171	1.690	0.21
Religion (Christianity vs. Islam)	1.045	0.614	1.780	0.49
Marital status (Never-married vs. Married)	2.184	0.706	6.752	0.13
Graduate level of education vs. Postgraduate level of education	1.773	0.182	17.270	0.53
Occupation (Employed vs. Unemployed)	1.412	0.501	3.976	0.35
COVID-19 Knowledge score	1.128	1.004	1.208	0.02

OR, odds ratio; CI, confidence interval.

The mean scores for KAB were correlated across the factors: knowledge of COVID-19 and attitudes were weakly positively correlated, *r*(25) = 0.132, *p* = 0.513. Knowledge of COVID-19 and behaviours was weakly positively correlated, *r*(25) = 0.34, *p* = 0.085. Finally, behaviours and attitudes were weakly positively correlated, *r*(25) = 0.26, *p* = 0.188. The correlations are weak, and the *p*-values are > 0.05; further regression analysis is not needed. Nevertheless, behaviours showed the tendency as the predictor of KAB (Table 7).

**TABLE 7:** Mean scores for KAB towards COVID-19.

Characteristics	Knowledge score (*n* = 12)		Attitudes (%)		Behaviours (%)	
	Mean	±SD	Mean	±SD	Mean	±SD
**Gender**						
Female	9.8	±2.4	75.7	±12.8	63.4	±20.8
Male	9.6	±2.3	73.3	±14.6	62.9	±21.4
**Age Group**						
16 to 20	9.8	±2.4	73.3	±13.2	65.0	±20.2
21 to 25	9.7	±2.2	75.3	±16.6	61.6	±22.7
26 to 30	9.5	±1.5	69.6	±8.90	69.9	±18.8
31 to 40	9.9	±2.9	96.3	±5.20	61.6	±22.7
41 to 50	6.5	±4.8	66.7	±23.6	38.9	±28.3
51 and above	7.9	±4.3	83.3	±13.6	55.6	±28.3
**Marital status**						
Married	9.2	±0.9	79.6	±15.8	54.7	±36.6
Never married	10.2	±10.7	73.7	±13.5	63.2	±20.7
**Education**						
Graduate	9.7	±2.3	73.4	±16.7	61.4	±21.3
Postgraduate	9.7	±2.1	83.7	±8.20	67.3	±19.6
Undergraduate	9.0	±5.4	73.8	±13.5	100	±0.00
**Religion**						
Christianity	9.8	±2.3	74.2	±12	74.2	±12.0
Islam	9.9	±2.4	75.1	±16.8	75.1	±16.8
Traditionalist	9.6	±2.3	100	±0.0	100	±0.00
**Occupation**						
Government employee	10.1	±13.1	85.2	±9.4	51.9	±22.9
Private sector worker	10.1	±12.1	88.1	±9.4	64.4	±24.6
Self employed	9.9	±2.7	73.9	±9.5	64.8	±22.3
Student	9.7	±2.3	74.8	±13.9	63.5	±20.9
Unemployed	10.0	±12.4	68.9	±12.3	62.8	±22.9
**Region of Resident**						
Northcentral	9.3	±3.1	70.1	±8.1	65.6	±15.5
Northeast	8.7	±3.2	76.7	±12.5	59.7	±23.2
Northwest	9.6	±2.3	81.8	±25.7	72.7	±12.9
Southeast	10.0	±13.1	52.4	±13.4	66.6	±13.5
Southsouth	9.2	±2.2	64.8	±30.2	57.4	±18.9
Southwest	9.8	±2.2	75.6	±12.8	63.0	±22.6

* , *p* < 0.05;

** , *p* < 0.01;

*** , *p* ≤ 0.001;

**** , *p* < 0.0001

SD, standard deviation.

## Discussion

This survey investigated the KAB of university students toward COVID-19, constituting the first such survey amongst university students in Nigeria. The study respondents were mainly young adults primarily located in the country’s southwestern region. There was an overall correct score of 78.7% (*n* = 863) on the knowledge of COVID-19, indicating that most of the respondents were knowledgeable about COVID-19. Most of them had a positive attitude towards the COVID-19 pandemic: 88.2% (*n* = 967) believed that COVID-19 would finally be successfully controlled, and 80% (*n* = 877) were confident that Nigeria could curb the spread of the virus. The high level of positive attitudes did not appear to translate into positive behaviours amongst all the students: over half of the respondents, 52.1% (*n* = 571), visited crowded places. However, most of them, 92.5% (*n* = 1014) wore masks when leaving home during the rapid first wave period of the COVID-19 outbreaks.

The findings of this study showed a high rate of COVID-19 knowledge amongst the respondents. This result was expected because this cross-sectional survey was conducted amongst educated university students who have access to information. A positive association between education level and COVID-19 knowledge scores was also noted. The positive correlation between university students and the knowledge of the signs and symptoms of disease shown in our study has previously been reported in a study by Ajilore et al. (2017) amongst university students, where they established a strong association between Ebola virus knowledge and college students’ attitudes towards the disease. Their findings suggested that college students’ knowledge of Ebola was high, even though they had misconceptions about the virus and there were gaps in their knowledge of the virus (Ajilore et al. 2017). A recent study in Nigeria amongst people living in the country’s North Central region also supported the findings of this current study, as they reported that the respondents were knowledgeable about the COVID-19 (Reuben et al. 2021). Other studies in Africa, Asia and Europe also reported high levels of knowledge of COVID-19 (Austrian et al. 2020; Huynh et al. 2020; Khan et al. 2020; Olapegba et al. 2020; Reuben et al. 2020; Saqlain et al. 2020; Zhong et al. 2020). The high level of COVID-19 knowledge recorded in this study could be attributed to the high level of education of the students, who have access to websites such as the Centers for Disease Control and Prevention (CDC) website and are familiar with using search engines to conduct research which requires health data for their work. As an extension, they could use these sites to access information to further their knowledge about the pandemic. In addition, the university was posting warnings on their websites and via bulk emails and WhatsApp messages.

There is also evidence that Ebola PSAs are generally accessible to students and that there is a strong association between Ebola knowledge and college students’ attitudes towards the disease. The findings suggest that college students’ knowledge of Ebola is high even though gaps in EVD-related knowledge and misconceptions persist. These findings have important implications for PSAs and prevention education initiatives for Ebola. In the future, PSAs and other prevention education initiatives must address misconceptions and pay particular attention to the false sense of security. There is a need for a proactive communication strategy to counter the growing belief that Ebola cannot resurface in Nigeria (Ajilore et al. 2017).

In this study, significant predictors of respondents’ knowledge were gender, age, educational level and marital status. The positive variance observed in this study amongst older and postgraduate respondents could be explained by the level of experience that comes with age and a higher education degree that increases intellectual prowess. A previous study showed that health knowledge acquisition is associated with familiarity and scholarly pursuits (Beier & Ackerman 2003). The higher knowledge of male respondents compared to female respondents could be due to the differences in digital media usage. Although both genders spent similar time on the internet, the type of usage differs, with female respondents having more interpersonal connections compared to male respondents who were orientated to acquiring information (Helsper 2010; Reiner et al. 2017). The study also found that male college students were more likely to look for news, read the online version of the school newspaper, and use the internet for political agendas than female students. In addition, the disproportionately high knowledge amongst single people compared to those who were married could be attributed to the comparatively high level of exposure to information via the internet amongst the youth, who constituted the unmarried respondents (Himawan et al. 2021).

The authors also reported a positive association between higher COVID-19 knowledge scores and a positive attitude. Over 80% of the respondents believed that Nigeria could control and ultimately curb the spread of COVID-19. Recent studies in other countries support the authors’ findings that optimistic attitudes can promote behaviours that help prevent the spread of the infection (Erfani et al. 2020; Qubais, Elbarazi & Barakat 2020; Zhong et al. 2020). The positive association between COVID-19 knowledge scores and positive attitude underscores the need for increased awareness and advocacy about COVID-19, which may eventually translate into positive behaviours and practices. Moreover, as reported previously, strong attitudes based on personal experience can easily influence behaviour (Ajilore et al. 2017; Clayton & Myers 2009). Furthermore, compliance with the preventive measures against the disease and sound knowledge of the signs and symptoms are associated with a positive attitude that ultimately influences behaviour (Ajilore et al. 2017; Crano & Prislin 2006).

The students’ positive attitude toward the control of COVID-19 could be related to the government’s unprecedented COVID-19 control measures to restrict movement in the country by shutting down the towns and cities and closing all borders. Secondly, the setting up of the presidential task force, which was replicated at the state level, also boosted the people’s confidence and precipitated a positive attitude. Thirdly, the residual memory of the country’s excellent handling and effective control of the Ebola outbreak within a brief period may have also instilled a sense of confidence amongst the respondents regarding the government’s ability to overcome the COVID-19 pandemic (Otu et al. 2018). This latter point is significant because an individual with a direct or indirect encounter or experience with a disease is more likely to develop strong preventive measures (Ajilore et al. 2017; Crano & Prislin 2006; Myers, Krawczyk & Agnello 2009).

The positive attitudes of most respondents towards COVID-19 have to a large extent translated into positive behaviours. The results of this study showed an overall 63.2% positive behaviours amongst the respondents. However, this percentage is lower compared to those reported by Olaimat et al. (2020) and Alzoubi et al. (2020) at 83.4% and 78%, respectively. Furthermore, despite about two-thirds of positive behaviours, some respondents, especially the young adults, indulged in risky behaviours, such as not wearing masks (7.5%) and going to crowded places. To increase compliance with wearing face masks, the government also recommended using locally made face masks (Adebowale 2020), as face masks are efficacious in reducing viral particles in the respiratory droplets (Leung et al. 2020; Razvi & Prasad 2020).

The high risk of going to a crowded place could be due to the young age of most of the respondents. A previous study reported that many young people do not understand the danger posed by COVID-19, claiming that only the elderly and people with compromised immune systems were dying from the disease (Kreitz 2020). In addition, previous studies have associated male adolescents with risk-taking behaviours (Cobey et al. 2013; Duell et al. 2018; Pawlowski, Atwal & Dunbar 2008; Zhong et al. 2020).

One of the major limitations of this study is that the findings are not representative of all university students in Nigeria, given that the sample was drawn from a single university. Although students’ age and gender were representative of demographics of a typical Nigerian university, the sample generally does not reflect the ethnic and socioeconomic diversity across the country. Another limitation is that not all the students would have access to the questionnaire if they had returned home at the time of the distribution of the questionnaire, as they may not have had internet or WiFi in their homes.

## Conclusion

Majority of the Nigerian university students who took part in the survey displayed knowledge of COVID-19, which translated into positive attitudes and compliance with safety practices, which are paramount in curbing the spread of the infection. The interaction between knowledge and attitudes leading to positive behaviours and adherence has been linked to how information was presented. Although this study was conducted before the development of vaccines, health advocacy activity participation, safe environments and strong relationships between students and caregivers could also play a role in influencing attitudes and behaviour.
